# *In vivo* analysis of *Drosophila* chondroitin sulfate biosynthetic genes

**DOI:** 10.1016/j.jbc.2025.110783

**Published:** 2025-10-07

**Authors:** Tomomi Izumikawa, Ayano Moriya, Eriko Nakato, Kako Yamamoto, Raiki Sano, Takuya Akiyama, Akiko Kinoshita-Toyoda, Hidenao Toyoda, Hiroshi Nakato

**Affiliations:** 1 Department of Genetics, Cell Biology, and Development, University of Minnesota, Minneapolis, Minnesota, USA; 2 Faculty of Pharmaceutical Sciences, Ritsumeikan University, Shiga, Japan; 3 Department of Biology, Indiana State University, Terre Haute, Indiana, USA

**Keywords:** chondroitin sulfate, Chsy, Csgalnact, Chpf, C4st, *Drosophila*

## Abstract

Chondroitin sulfate (CS) is an evolutionarily conserved class of glycosaminoglycans and is found in most animal species. Previous studies of CS-deficient *Drosophila* models, *Chondroitin sulfate synthase* (*Chsy*), and *Chondroitin polymerizing factor* (*Chpf*) mutants demonstrated the importance of CS in the structural integrity of the basement membrane and organ shape maintenance. However, biosynthetic mechanisms of *Drosophila* CS remain to be elucidated. To investigate the CS biosynthesis in *Drosophila*, we generated mutants for two additional biosynthetic enzyme genes, *CS N-acetylgalactosaminyltransferase* (*Csgalnact*) and *CS 4-O sulfotransferase* (*C4st*), using CRISPR–Cas9 mutagenesis. *Csgalnact*-null mutants show moderate changes in CS biosynthesis, including reduced CS in the larval brain and altered CS chain length. We found that this gene is dispensable for normal viability and morphogenesis. On the other hand, *C4st* mutants show more severe defects, including a high level of lethality and a folded wing phenotype. The *C4st* mutation not only eliminates CS sulfation but increases production of unsulfated chondroitin, suggesting the existence of a compensatory feedback mechanism. Both *Csgalnact* and *C4st* mutants show impaired adult negative geotaxis behavior, consistent with the role of CS proteoglycan in the neuromuscular systems. Our study revealed unique and poorly understood features of invertebrate CS biosynthesis and provides novel *in vivo* toolsets to investigate CSPG functions in development.

Chondroitin sulfate (CS), an evolutionarily conserved class of glycosaminoglycans, is covalently linked to specific core proteins to form CS proteoglycans (CSPGs) that are distributed on the cell surface and in the extracellular matrix. CSPGs exert a wide variety of biological functions in developmental and physiological processes, such as cytokinesis, morphogenesis, maintenance of stem cell pluripotency, and neuroplasticity (1, 2). Aberrant CSPG biosynthesis underlies various pathological conditions, including skeletal disorders and cancer metastasis (3, 4).

The polymerization and modification of CS chains are tightly controlled by glycosyltransferases and sulfotransferases (5). CS and heparan sulfate are attached to specific serine residues of core proteins through the common linkage tetrasaccharide, GlcAβ1-3Galβ1-3Galβ1-4Xylβ1-*O*-Ser. After the linkage region is formed, CS chain is synthesized as repeating disaccharide [-4GlcAβ1-3GalNAcβ1-]_n_ by alternate addition of GalNAc and GlcA residues. CS chain initiation requires *N*-acetylgalactosaminyltransferase-I (GalNAcT-I) activity, whereas chain elongation needs *N*-acetylgalactosaminyltransferase-II (GalNAcT-II) and glucuronosyltransferase-II activities.

In humans, six homologous glycosyltransferases responsible for biosynthesis of the CS backbone have been identified (5, 6, 7, 8, 9, 10, 11, 12) (Fig. 1*A*). Based on their enzymatic activity *in vitro*, members of this family were designated as CHSY1 (chondroitin sulfate synthase 1, chondroitin synthase 1, ChSy-1, or CSS1), CHSY3 (ChSy-2 or CSS3), CHPF (chondroitin polymerizing factor, ChPF, or CSS2), CHPF2 (ChSy-3 or CSGlcAT), CS *N*-acetylgalactosaminyltransferase-1 (CSGALNACT1) (ChGn-1, or CSGalNAcT-1), and CS *N*-acetylgalactosaminyltransferase-2 (CSGALNACT2) (ChGn-2 or CSGalNAcT-2). The three enzymes, CHSY1, CHSY3, and CHPF2, possess dual glycosyltransferase activities, glucuronosyltransferase-II and GalNAcT-II. The chain elongation takes place by the action of a complex consisting of any two of four proteins—CHSY1, CHSY3, CHPF, and CHPF2 (5, 7). CSGALNACT1 (ChGn-1 or CSGalNAcT-1) and CSGALNACT2 (ChGn-2 or CSGalNAcT-2) have both GalNAcT-I and GalNAcT-II activities and are involved in CS chain initiation and elongation (8, 9, 10, 12).

**Figure 1:**
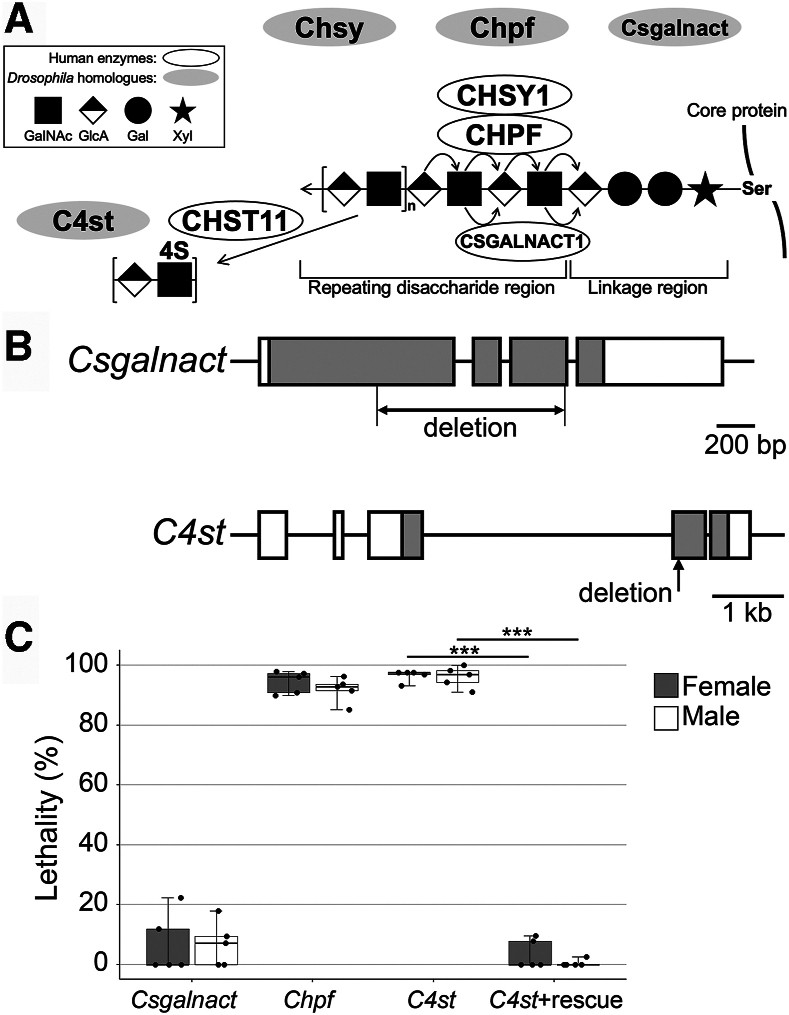
**Generation of *Csgalnact and C4st* mutant alleles.***A*, human and *Drosophila* CS biosynthetic enzymes. CS chains are covalently attached to specific serine residues of the core protein through a common linkage tetrasaccharide (the linkage region). In this schematic, four of six human glycosyltransferase paralogs responsible for CS backbone biosynthesis are shown by *white ovals*. CS polymerization is catalyzed by a heterodimeric complex composed of two of the four molecules, CHSY1, CHSY3, CHPF, and CHPF2. In this schematic, the CHSY1–CHPF complex is shown. CSGALNACT1 and CSGALNACT2 possess both GalNAcT-I and GalNAcT-II activities and participate in CS chain initiation and elongation. Sulfation at the C4 position of GalNAc (4S) is catalyzed by a 4-*O* sulfotransferase, encoded by CHST11. *Drosophila* enzymes, Chsy, Chpf, Csgalnact, and C4st, homologous to human CHSY1, CHPF, CSGALNACT1, and CHST11, respectively, are shown by *gray ovals*. Only human enzymes corresponding to these *Drosophila* homologs are depicted in this figure, and other human CS biosynthetic enzymes are not shown. *B*, a schematic of the *Csgalnact and C4st* locus. Mutant alleles were generated by the CRISPR–Cas9-mediated gene editing, which induced a large deletion of 1018 bp in the locus (*Csgalnact*^*212*^) and a single base pair deletion the *C4st* locus (*C4st*^*340*^). Based on the resultant sequences, both alleles are considered to be null. *C*, lethality of *Csgalnact*, *Chpf*, *and C4st* alleles. The lethality of *C4st* mutants was completely rescued by *UAS-C4st* expression with a ubiquitous Gal4 driver, *act-Gal4* (C4st + rescue: *C4st*^*340*^*act-Gal4/C4st*^*340*^*UAS-C4st*). The lethality rates of females or males for each genotype were calculated by five independent sets of experiments. Each experiment was performed for each sex, and >250 flies per set were counted. *Boxes* indicate the 25th to 75th percentiles, and the median is marked with a *line*. The *whiskers* extend to the highest and lowest values within 1.5 times the interquartile range. ∗∗∗*p* < 0.001 (two-sided, unpaired *t* test). CS, chondroitin sulfate; GalNAcT-I, *N*-acetylgalactosaminyltransferase-I; GalNAcT-II, *N*-acetylgalactosaminyltransferase-II.

Biosynthesis of CS–chondroitin in invertebrates has been extensively studied using a genetically tractable model, the nematode *Caenorhabditis elegans* (*C. elegans*). In this species, only three genes are known to function in CS biosynthesis: *squashed vulva-5* (*sqv-5*, the *CHSY* homolog), *mig-22* (*PAR2.4*, the *CHPF* homolog), and *chst-1* (the homolog of carbohydrate sulfotransferase 11 [*CHST11*], human CS 4-*O* sulfotransferase) (2, 13, 14, 15, 16). *C. elegans* has no homolog of CSGALNACT. As Sqv-5 possesses both GalNAcT-I and -II activities (13, 14), it is believed to catalyze both the initiation and elongation of CS–chondroitin chains. *C. elegans* produces a substantial amount of unsulfated chondroitin, and it was initially believed to synthesize only chondroitin but not CS. However, a low level of sulfation was detected in later studies, and *chst-1* was identified as a *C. elegans* CS 4-*O* sulfotransferase (15, 16). Nevertheless, functional importance of CS sulfation in *C. elegans* remains to be determined. This unique CS–chondroitin biosynthetic system in *C. elegans* raised a few important questions. First, the lack of *C. elegans* homolog of CSGalNAcT raises questions about the biological significance of this enzyme: Is CSGalNAcT required for CS biosynthesis in other invertebrate species? How did this molecule emerge during evolution and become an essential component of the CS biosynthetic machinery? Second, Is CS sulfation important for CSPG functions in invertebrates?

The *Drosophila* genome contains at least four CS biosynthetic enzyme genes, *Chsy*, *Chpf*, *Csgalnact*, and *CS 4-O sulfotransferase* (*C4st*), which are the *Drosophila* homologs of human *CHSY*, *CHPF*, *CSGALNACT1*, and *CHST11*, respectively (Fig. 1*A*). We previously generated and characterized mutants in *Chsy* and *Chpf* (2, 17, 18). Analyses using the adult ovary as a model system revealed that CS is required for normal mechanical properties and structural integrity of the basement membrane. In addition, the function of the ovarian muscle sheath is impaired in the absence of CS. As a result, *Chsy* mutants fail to maintain gross organ structure during aging. Furthermore, we demonstrated that both *Chsy* and *Chpf* are required to properly regulate intestinal stem cell proliferation in the adult midgut (18). Regarding sulfation of *Drosophila* CS, we have previously shown that *Drosophila* has two clearly separable populations of CSPGs: one with 4-*O*-sulfated CS and another with unsulfated chondroitin (19). However, the developmental functions of 4-*O*-sulfated CS remain to be determined.

In this study, to understand the molecular mechanisms underlying CS biosynthesis in *Drosophila*, we isolated null mutants for two additional genes of the CS biosynthetic/modifying enzymes, *Csgalnact* and *C4st*. These mutations allowed us to uncover the biological significance of these molecules during *Drosophila* development.

## Results

### Generation of mutants for CS biosynthetic genes

In addition to *Chsy* and *Chpf*, the *Drosophila* genome contains an additional Chsy family gene: *Csgalnact* (Fig. 1*A*). *Csgalnact* is the *Drosophila* homolog of human *CSGALNACT1* and *CSGALNACT2*, which are believed to catalyze chain initiation and elongation, respectively (12, 20, 21, 22). Using CRISPR–Cas9 mutagenesis, we generated *Csgalnact*^*212*^, which has a large deletion (1018 bp) in the protein coding sequence spanning from exon 1 to 3 (Figs. 1*B* and S1). This results in a truncated protein at Val207, which lacks the domain critical to enzymatic activity (9, 10).

In addition to glycosyltransferases, sulfotransferases are another class of key enzymes critical to the production of functional CS (5, 23). In *Drosophila*, a protein encoded by CG31743 exhibits a clear sequence similarity to human CS sulfotransferase, CHST11 (Fig. 1*A*). We showed that RNAi knockdown against CG31743 abolished the epitope of anti-CS antibody (LY111) (19). This antibody is known to recognize highly sulfated CS structures, such as 4-*O*-sulfated CS, GlcAβ1-3GalNAc(4S) (24). Thus, a sulfotransferase encoded by CG31743 is required to generate a sulfated CS structure (the LY111 epitope) and is believed to be the *Drosophila* CS 4-*O* sulfotransferase (19, 25). Therefore, this gene is referred to as *C4st* in this article. A mutant allele in this locus, *C4st*^*340*^, was generated by a single base pair deletion at Ser33, leading to a frame shift that eliminated the sulfotransferase domain (Figs. 1*B* and S2) (26, 27). Thus, both *Csgalnact*^*212*^ and *C4st*^*340*^ alleles we isolated in this study lack functional domains and are considered amorphic (Figs. S1 and S2).

### *Drosophila* CS biosynthetic gene mutants

As a first step of characterization of the CS biosynthetic gene mutants, we first determined their lethality. Our previous study showed that the *Chsy* null mutants show a high level of lethality around 95% in both sexes (17). We found that the lethality of *Chpf* and *C4st* mutants is comparable to *Chsy* (Fig. 1*C*), ranging 92% to 97% in both females and males. The lethality of *C4st* mutants was completely rescued by *UAS-C4st* expression with a ubiquitous Gal4 driver, *act-Gal4* (*C4st* + rescue: *C4st*^*340*^ *act-Gal4/C4st*^*340*^ *UAS-C4st*). On the other hand, *Csgalnact* showed a significantly lower level of lethality, with an average of 6.8% (females) and 6.7% (males) (Fig. 1*C*).

**Figure 2:**
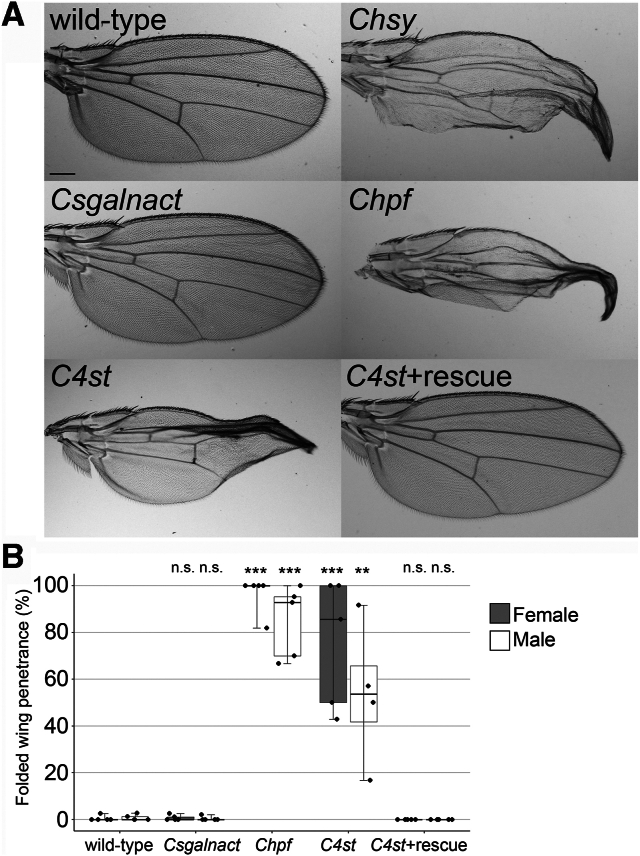
**Wing maturation defects of CS biosynthetic gene mutants.***A*, adult wings are shown for wildtype, *Chsy*, *Csgalnact*, *Chpf*, *and C4st* mutants. *Chsy*, *Chpf, and C4st* mutants show a wing maturation defect. Expression of *C4st* from a *UAS-C4st* transgene fully rescues the wing maturation defect of *C4st* mutants (C4st + rescue: *C4st*^*340*^*act-Gal4/C4st*^*340*^*UAS-C4st*). *B*, penetrance of the wing folding phenotype for mutant females and males. More than four sets of experiments were performed for each sex; over 250 flies per set were counted. *Boxes* indicate the 25th to 75th percentiles, and the median is marked with a *line*. The *whiskers* extend to the highest and lowest values within 1.5 times the interquartile range. ∗∗*p* < 0.01; ∗∗∗*p* < 0.001; ns, not significant (one-way ANOVA with Dunnett's test). The scale bar represents 200 μm. CS, chondroitin sulfate.

We previously reported that a large fraction of *Chsy* adult survivors (approximately 72–79%) showed a folded wing phenotype, indicating a wing maturation defect (17). The wing maturation process is the last step of wing development (28, 29). This process includes apoptosis of wing epithelial cells, absorption of hemolymph and cellular debris from the wing into the body, and adhesion of the dorsal and ventral cuticle sheets. A failure of this process typically results in folded wings. No gross morphological defect (*e.g*., wing margin formation and venation) was observed in *Chsy* mutant wings (17). These observations suggested that CS is mainly required for normal wing maturation but not overall wing patterning. This idea was supported by the newly isolated mutants. *Chpf*^*424*^ and *C4st*^*340*^ show a folded wing phenotype that are indistinguishable from *Chsy* wings (Fig. 2*A*). The penetrance of the wing phenotype of *Chpf*^*424*^ was 85% to 97% in both sexes, which is comparable to *Chsy* mutants (Fig. 2*B*). *C4st*^*340*^ showed this phenotype with a somewhat reduced penetrance (approximately 53–76%). *C4st* expression by *act-Gal4* fully rescued the folded wing phenotype of *C4st* mutants (C4st + rescue in Fig. 2, *A* and *B*). These observations suggest that 4-*O* sulfated CS functions in the wing maturation process, but unsulfated CS may retain a residual activity. In contrast, homozygotes of *Csgalnact*^*212*^ rarely showed this phenotype (penetrance <1%, Fig. 2, *A* and *B*). Thus, we found that *Chpf* and *C4st* mutants show modest-to-high levels of lethality and a defect consistent with the previously known phenotype for CS deficiency. Interestingly, however, *Csgalnact*-null mutants do not show any obvious morphological phenotypes.

### CS polymerization in CS biosynthetic mutants

**Figure 3:**
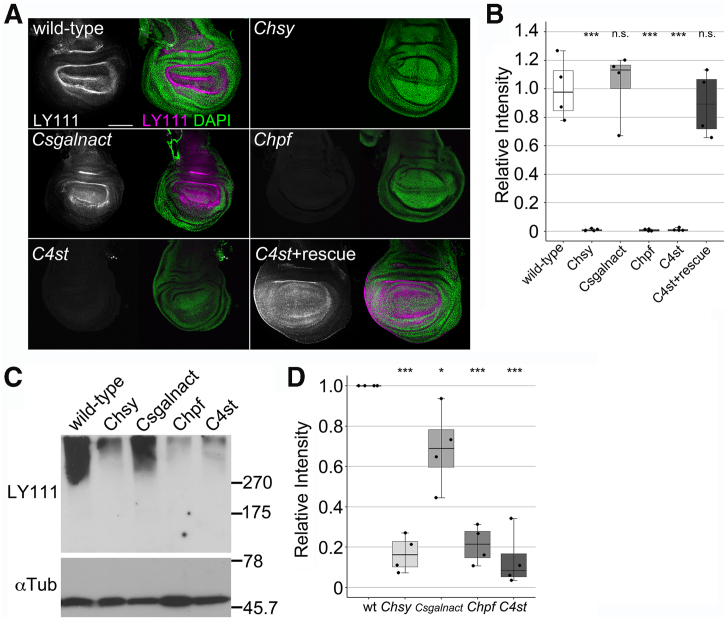
**CS production in CS biosynthetic gene mutants.***A*, immunostaining of wing discs from wildtype, *Chsy*, *Csgalnact*, *Chpf*, and *C4st* mutant third instar larvae with anti-CS antibody (LY111, *magenta*). The loss of the LY111 epitope in *C4st* mutants was completely rescued by *UAS-C4st* expression (C4st + rescue: *C4st*^*340*^*act-Gal4/C4st*^*340*^*UAS-C4st*). The discs were counter stained with DAPI (*green*). *B*, quantification of anti-CS staining. The anti-CS intensity was normalized by DAPI, and the average value in wildtype was defined as 1.0. *C*, immunoblot analysis of CS biosynthetic gene mutants. Protein extracts from wildtype, *Chsy*, *Csgalnact*, *Chpf*, and *C4st* adult flies were subjected to immunoblot analysis using anti-CS antibody (LY111). Anti-αTubulin antibody was used as the internal control. We believe that a signal at the top of the gel in Figure 3*C* is a nonspecific background as this has been observed in immunoblot analyses using LY111 with crude *Drosophila* homogenate samples in our previous studies (17, 19). *D*, quantification of immunoblot analysis. This graph summarizes data from four independent experiments. *Boxes* indicate the 25th to 75th percentiles, and the median is marked with a *line*. The *whiskers* extend to the highest and lowest values within 1.5 times the interquartile range. ∗*p* < 0.05; ∗∗∗*p* < 0.001; ns, not significant (one-way ANOVA with Dunnett's test). The scale bar represents 100 μm. CS, chondroitin sulfate; DAPI, 4′,6-diamidino-2-phenylindole.

To determine if CS production is affected in the CS biosynthetic mutants, we performed immunohistochemical analysis using anti-CS antibody (LY111). As we have shown previously, the LY111 signal is detected in the basement membrane throughout the wildtype wing disc (Fig. 3*A*; (19)). We found that the LY111 epitope was lost in the discs from *Chsy*, *Chpf*, and *C4st* mutants (Fig. 3, *A* and *B*). In contrast, the LY111 signal in *Csgalnact* wing discs was indistinguishable from the wildtype control. The LY111 epitope in *C4st* mutant wing discs was fully recovered by *C4st* expression by *act-Gal4* (*C4st* + rescue in Fig. 3, *A* and *B*).

We next analyzed CSPGs in the CS biosynthetic mutants by immunoblot analysis using LY111. This antibody detects high molecular weight CSPGs (>200 kDa) as smear bands in wildtype protein extracts (Fig. 3*C*; (17, 19)). As has been shown previously, these smear bands were undetectable in extracts prepared from *Chsy* adults (Fig. 3*C*; (17). Consistent with the immunohistochemistry data, the smear bands are absent in the extract from *Chpf* and *C4st* mutants. In protein extracts from *Csgalnact* mutants, we detected the smear bands (Fig. 3*C*). However, the signal intensity of the band in the *Csgalnact* mutant sample was slightly lower than wildtype (Fig. 3, *C* and *D*).

Together, the LY111 epitope was undetectable in *Chsy*, *Chpf*, and *C4st* mutants, but CS production appears to occur in the *Csgalnact* null mutants. This shows a striking contrast with the importance of CSGALNACT1 for the CS biosynthesis in mammals. Nevertheless, it is consistent with the phenotypic analyses, showing the absence of obvious defects in *Csgalnact* mutants.

### Glycosyltransferase activity of *Csgalnact*

As we did not observe any morphological and biochemical abnormality in *Csgalnact* mutants, we examined enzymatic activity of Csgalnact. We expressed a soluble form of Csgalnact protein, lacking the putative cytoplasmic and transmembrane domains, with a FLAG epitope tag in 293T cells. As a positive control, an equivalent construct of human CSGALNACT1 was used (8, 22, 30). Both human and *Drosophila* recombinant enzymes were recovered from the conditioned media and purified using anti-FLAG affinity agarose. The recovery of both proteins was confirmed by immunoblot analysis using an anti-FLAG antibody (Fig. S3).

We assayed GalNAc-transferase activity of the recombinant proteins using 4-methylumbelliferyl-β-d-glucuronic acid (GlcA-MU) as the sugar acceptor (8, 22, 30). The products of the reactions on GlcA-MU were isolated by gel filtration and fluorometrically quantified (Table 1). We found that *Drosophila* Csgalnact indeed possesses the GalNAc-transferase activity while its relative activity is significantly lower than human CSGALNACT1 (approximately 1:40, Table 1). Previous studies of glycosyltransferase activity in *Drosophila* enzymes also reported that these molecules exhibited much lower activities *in vitro* (31). It is possible that some assay conditions, such as expression in mammalian cell lines (293T cells) and the incubation temperature (37 °C), may not be fully optimized to *Drosophila* enzymes. In any case, we concluded that *Csgalnact* encodes an active GalNAc transferase.

**Table 1:** GalNAc transferase activities of fusion proteins secreted into the culture medium by the transfected 293T cells

CSGalNAcT ortholog	GalNAc transferase activity nmol/ml medium/h
Human CSGALNACT1 (n = 3)	381.1 ± 11.9
*Drosophila* Csgalnact (n = 3)	9.5 ± 1.1

The values are given as nmol/ml medium/h and represent mean ± SD from three biological replicates. GlcA-MU was used as an acceptor substrate.

### CS disaccharide structures in CS biosynthetic mutants

**Figure 4:**
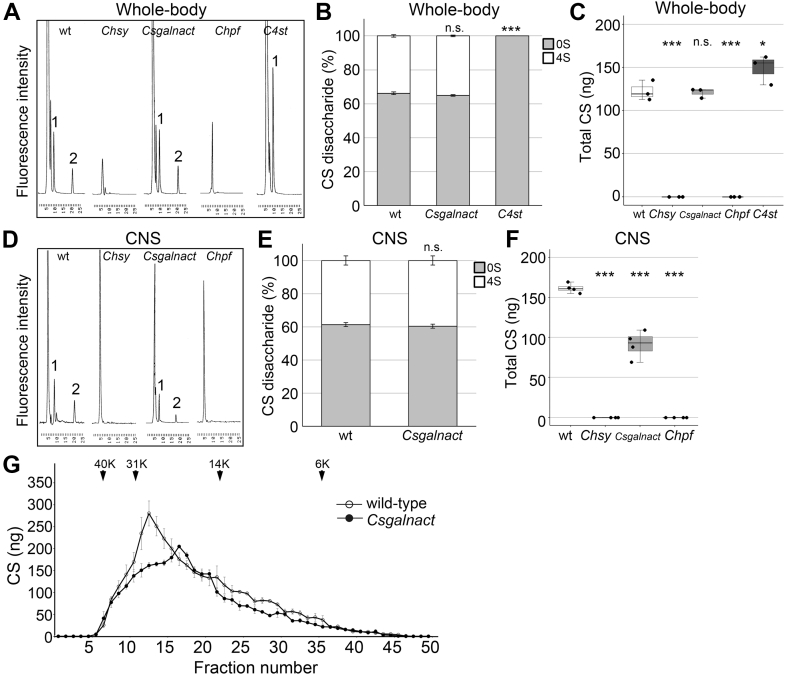
**CS structural analyses of CS biosynthetic gene mutants.***A*–*F*, CS disaccharide analysis. After CS was completely digested with chondroitinase ABC, the resultant disaccharide species were separated by reversed-phase ion-pair chromatography with a postcolumn detection system. Chromatograms of unsaturated disaccharides from adult flies (*A*) or third instar larval CNS (*D*) with indicated genotypes. Peaks for the two disaccharides ΔDi-0S (1) and ΔDi-4S (2) are marked. Based on these results, the disaccharide ratios, the percentages for ΔDi-0S (*gray*, 0S) and ΔDi-4S (*white*, 4S), are shown as stacked bar graphs (*B* and *E*). Total CS (ng/mg dry tissue) prepared from adult whole-body samples (*C*) or third instar larval brains (*F*) is compared between indicated genotypes. These data are also shown as tables (Tables 2 and 3). *G*, CS chain length changes in *Csgalnact* mutants. CS fractions were separated by gel-filtration chromatography on a Superose 12 10/300 GL column. CS samples in individual fractions were digested with chondroitinase ABC and analyzed by HPLC. The amounts of unsaturated disaccharides were calculated based on fluorescence intensity. Samples from wildtype (*open circle*) and *Csgalnact* mutants (*closed circle*) are shown. *Arrowheads* indicate the size of molecular mass standards in kilodalton. These data represent the average of two independent experiments that showed essentially the same profiles. *Boxes* indicate the 25th to 75th percentiles, and the median is marked with a *line*. The *whiskers* extend to the highest and lowest values within 1.5 times the interquartile range. ∗*p* < 0.05; ∗∗∗*p* < 0.001; ns, not significant (one-way ANOVA with Dunnett’s test and two-sided, unpaired *t* test). CS, chondroitin sulfate; CNS, central nervous system.

We next examined structures of CS isolated from the CS biosynthetic mutants by disaccharide analysis (Fig. 4*A* and Table 2). Briefly, CS specimens were isolated from adult whole bodies of wildtype and mutant animals. After complete digestion of CS with chondroitinase ABC, the resulting disaccharide species were separated using reversed-phase ion-pair chromatography. This analysis detects two CS disaccharide peaks: ΔDi-0S (marked as 1 in Fig. 4*A*) and ΔDi-4S (marked as 2). As we have reported previously, both peaks are undetectable in *Chsy* mutants (17). Similarly, *Chpf* mutation eliminates both ΔDi-0S and ΔDi-4S (Fig. 4*A* and Table 2). Thus, consistent with the lack of the LY111 epitope in the immunoblot and immunohistochemical assays of *Chsy* and *Chpf* mutants, the disaccharide analysis confirmed the absence of CS in these mutants.

**Table 2:** Percentages of 4-*O* sulfated disaccharide in CS derived from wildtype and mutant adults

Genotype	4-*O* Sulfated disaccharide (%)	Total CS (ng/mg dry tissue)
Wildtype (n = 3)	33.8 ± 0.8	123 ± 9.4
*Chsy* (n = 3)	—	ND^a^
*Csgalnact* (n = 3)	35.1 ± 0.5 (ns)	121 ± 4.7 (ns)
*Chpf* (n = 3)	—	ND^a^
*C4ST* (n = 3)	0^a^	149 ± 14.0^b^

The values are given as mol % of 4-*O* sulfated disaccharide (ΔDi-4S) and represent mean ± SD from three biological replicates. One-way ANOVA with Dunnett's test was used to calculate *p* values.

a *p* < 0.001

b *p* < 0.05

Disaccharides obtained from *C4st* mutants lack ΔDi-4S (Fig. 4*A* and Table 2). Instead, we detected a massive increase in the ΔDi-0S level compared with wildtype (Fig. 4, *A* and *B*), leading to an elevated amount of total CS (Fig. 4*C* and Table 2). This finding demonstrates that the lack of the LY111 epitope in *C4st* mutants reflects the loss of sulfated residues, not the absence of chondroitin polymer. This result is consistent with the predicted function of this molecule. In addition, it is likely that *C4st* ablation induces a compensatory increase in CS production, resulting in a high level of unsulfated chondroitin.

In *Csgalnact* mutant whole-body samples, total amount of CS and the disaccharide composition were similar to those in wildtype (Fig. 4, *A*–*C* and Table 2). This observation suggests that *Drosophila* Csgalnact plays a relatively minor role in the CS biosynthesis in this species. For example, it may have a redundant function with Chsy in a limited number of cells. In fact, high-throughput gene expression data (FlyAtlas Anatomical Expression Data) indicate that *Csgalnact* is almost exclusively expressed in the larval, pupal, and adult central nervous system (CNS). We therefore examined the CS disaccharide profile using the third instar larval brain samples from wildtype and *Csgalnact* mutants. We found that the total amount of CS was significantly decreased (approximately 40% reduction) in *Csgalnact* mutant CNS (Fig. 4, *D* and *F*, Table 3). The disaccharide composition was unaffected (Fig. 4, *D* and *E*, Table 3). This result supports the idea that *Csgalnact* contributes to CS biosynthesis in the developing CNS.

**Table 3:** Percentages of 4-*O* sulfated disaccharide in CS derived from third instar larval brains

Genotype	4-*O* Sulfated disaccharide (%)	Total CS (ng/mg dry tissue)
Wildtype (n = 4)	38.0 ± 1.2	161.6 ± 5.1
*Chsy* (n = 4)	—	ND^a^
*Csgalnact* (n = 4)	39.7 ± 2.8 (ns)	91.2 ± 14.8^a^
*Chpf* (n = 4)	—	ND^a^

The values are given as mol % of 4-*O* sulfated disaccharide (ΔDi-4S) and represent mean ± SD from four biological replicates. Two-sided, unpaired *t* test and one-way ANOVA with Dunnett's test were used to calculate *p* values.

a *p* < 0.001

When *Csgalnact* is involved in CS production, does it require the function of Chsy and/or Chpf? As stated previously, CS was not detectable in the whole-body specimens from *Chsy* and *Chpf* mutants (Fig. 4, *A* and *C*, Table 2). We confirmed that CS is undetectable in the larval CNS in these mutants (Fig. 4, *D* and *F*, Table 3). Therefore, even in the presence of Csgalnact, an active glycosyltransferase involved in CS biosynthesis, both Chsy and Chpf are still required for CS production. This indicates that Csgalnact cannot perform a specific role that replaces Chsy or Chpf. For example, Csgalnact may contribute to chain initiation, whereas chain elongation may need the activity of both Chsy and Chpf.

### CS chain length in *Csgalnact* mutants

A previous study showed that mammalian *CSGALNACT1* affects the CS chain length (32). We therefore examined the length of CS chains obtained from *Csgalnact* mutants. After crude CS samples were fractionated by gel-filtration chromatography on a Superose 12 10/300 GL column, CS in each fraction was digested with chondroitinase ABC. The amount of the resulting disaccharides was quantified. This analysis of wildtype samples revealed a peak of high-molecular weight CS chains around 30 kDa (Fig. 4*G*). This high-molecular weight fraction was significantly reduced in *Csgalnact* mutants, with the highest peak shifting to a smaller size (Fig. 4*G*). The chromatogram patterns of shorter CS chains were similar between wildtype and *Csgalnact* mutants. The reduction of long CS chains in the mutants is consistent with the idea that this enzyme is involved in CS chain elongation.

### Behavioral phenotypes of CS biosynthetic mutants

We recently demonstrated that *Chsy* mutants exhibited abnormal morphology and compromised function of the ovarian muscle sheath (17). Furthermore, our observation that *Csgalnact* contributes to the CS production in the CNS suggests that CS may have a role in the neuromuscular system. To test this idea, we examined the effects of CS biosynthetic gene mutations on larval and adult behaviors *via* two behavioral assays.

**Figure 5:**
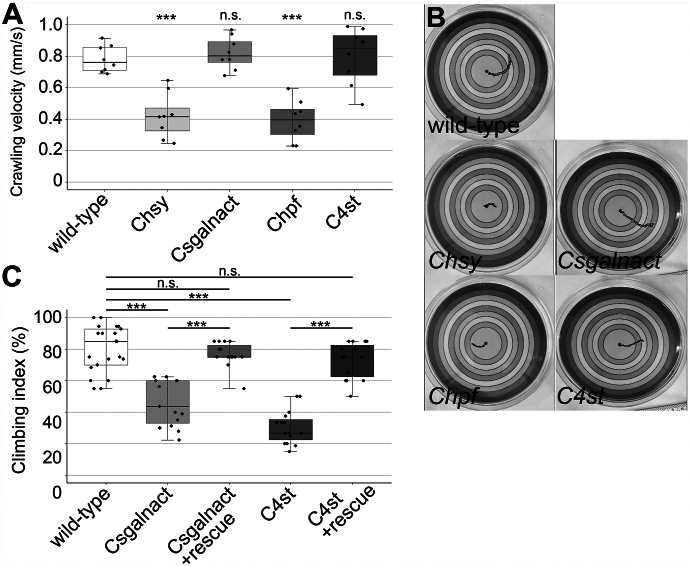
**Larval and adult behavioral phenotypes of CS biosynthetic mutants.***A* and *B*, larval locomotion assay. Crawling velocity (*A*) and traces of locomotion path (*B*) of third instar larvae of indicated genotypes. The larval crawling was monitored for 45 s (n = 8 larvae/genotype). *C*, negative geotaxis assay. The climbing ability (climbing index) of adult flies with indicated genotypes was quantified as number of animals that reached the top of a glass cylinder (10 cm) in 15 s. Twenty flies were assessed in an assay, and the test was repeated three times. More than 120 flies were tested for each genotype, and the mean percent value (±SEM) was calculated for at least six experiments. The climbing defects of *Csgalnact* and *C4st* mutants were fully rescued by the expression of *UAS-Csgalnact* (Csgalnact + rescue: *Csgalnact*^*212*^*act-Gal4/Csgalnact*^*212*^*UAS-Csgalnact*) or *UAS-C4st* (C4st + rescue: *C4st*^*340*^*act-Gal4/C4st*^*340*^*UAS-C4st*), respectively. *Boxes* indicate the 25th to 75th percentiles, and the median is marked with a *line*. The *whiskers* extend to the highest and lowest values within 1.5 times the interquartile range. ∗∗∗*p* < 0.001; ns, not significant (one-way ANOVA with Dunnett's test and two-sided, unpaired *t* test).

In one assay, we examined the larval locomotion of the CS biosynthetic mutants (33). Movement of an individual third instar larva on an agar plate was tracked at 1-s intervals to measure the velocity and path (distance traveled) of larval crawling. This assay showed that both velocity and path were significantly reduced in CS-deficient animals, *Chsy* and *Chpf* mutants, compared with wildtype (Fig. 5, *A* and *B*). On the other hand, these values in *Csgalnact* and *C4st* mutants were comparable to the wildtype control, showing that these two genes are dispensable for normal larval locomotion. Our observations indicate that important roles of unsulfated chondroitin in neural and/or muscular systems required for larval crawling.

We next asked if *C4st* and *Csgalnact* mutations affect adult behaviors. Negative geotaxis assay (or climbing assay) utilizes the natural upward climbing behavior of *Drosophila* adult flies and is widely used to evaluate animals' neuronal and motor functions (34, 35). The climbing assay revealed that *Csgalnact* mutants show a climbing deficit (Fig. 5*C*). As *Csgalnact* is predominantly expressed in the CNS, this result suggests that reduced CS in the mutant CNS impairs the neural function required for normal climbing behavior. We found that *C4st* mutants were more severely affected (Fig. 5*C*). Therefore, *C4st* mutation may have a more profound impact on the neural function than *Csgalnact*. Alternatively, 4-*O* sulfation of CS may have additional roles in muscle development and/or activities. The defective climbing ability of *Csgalnact* and *C4st* mutants was completely rescued by expression of respective genes by *act-Gal4* (*Csgalnact*^*212*^ *act-Gal4/Csgalnact*^*212*^ *UAS-Csgalnact* and *C4st*^*340*^ *act-Gal4/C4st*^*340*^ *UAS-C4st*) (Fig. 5*C*).

## Discussion

In humans, loss of *CHSY1* causes Temtamy preaxial brachydactyly syndrome, of which major features include limb malformations, short stature, and delayed motor and mental development (36, 37). Human diseases associated with other CS biosynthetic enzymes show dwarfism, skeletal dysplasia, and malformation of digits (38, 39). Thus, in vertebrates, the major consequence of impaired CS biosynthesis is defective skeletal system development and function. On the other hand, as CS exists in more primitive animal species with no cartilage or bone, CS must originally have nonskeletal functions. Powerful genetic studies in *C. elegans* revealed essential roles of CS–chondroitin in development and morphogenesis (2, 13, 14). At the same time, the lack of CSGalNAcT in *C. elegans* raised a question about the significance of this enzyme for CS production in invertebrate species. In addition, the scarceness of CS sulfation in *C. elegans* raised another question: what biological phenomena require CS sulfation, and what occurs in the absence of sulfation? To fill this knowledge gap, we isolated two novel mutations in the *Drosophila* CS biosynthetic machinery, *Csgalnact* and *C4st*.

We found that *Drosophila* Csgalnact plays a relatively minor role in CS biosynthesis in this species. High-throughput data show brain-specific expression of *Csgalnact* gene, and its null mutants show modest phenotypes, including reduced CS in the larval CNS and impaired adult negative geotaxis behavior, consistent with its function in the nervous system. We found that *Csgalnact* is dispensable for CS production outside the CNS, wing maturation, and larval locomotion. These observations suggest that *Drosophila* Chsy possesses both GalNAcT1-I and -II activities, as shown for *C. elegans* Sqv-5, so that it can catalyze CS chain initiation without Csgalnact. Specific molecular functions of *Csgalnact* in the CNS, such as neural network formation and/or maintenance, remain to be elucidated.

The molecular mechanism of glycosaminoglycan chain length control is poorly understood and is currently an important topic of study (20, 32, 40, 41, 42). We found that *Csgalnact* affects CS chain length. Gel-filtration chromatography showed that a long-chain fraction is significantly reduced in *Csgalnact* mutants. The pattern of short chains was not obviously affected. Thus, this enzyme is likely to be involved in CS chain elongation. It is unknown at this point whether this enzyme participates in chain initiation.

*C4st* mutants show more severe defects than *Csgalnact*, including a high level of lethality, a wing maturation defect, and reduced adult-negative geotaxis activity. Thus, CS sulfation plays a significant role in *Drosophila* development and physiology. The *C4st* mutant phenotypes are clearly less severe compared with those of *Chsy* and *Chpf* mutants. We have previously shown that *Drosophila* has two populations of CSPGs with 4-*O* sulfated CS chains and with unsulfated chondroitin (19). Comparison of the detailed phenotypes between *C4st* and *Chsy*/*Chpf* mutants will be able to define the functions of CS *versus* chondroitin. It is important to note that *C4st* mutant CS showed not only a lack of ΔDi-4S but also an increased level of ΔDi-0S. This indicates the existence of a possible compensatory feedback system to minimize the consequence of this mutation.

## Experimental procedures

### Drosophila strains

Oregon-R was used as a wildtype control. Flies were raised on a standard cornmeal fly medium at 25 °C unless otherwise indicated. We previously generated *Chsy*^*2*^ and *Chpf*^*424*^, null alleles for respective genes, and were used in this study (17, 18). *Csgalnact*^*212*^ and *C4st*^*340*^ mutant strains were generated in this study by CRISPR–Cas9-mediated nonhomologous end joining as previously described (17, 43). *UAS-Csgalnact* and *UAS-C4st* were generated and used for rescue experiments in this study. sgRNA sequences targeting these genes chosen using CRISPR Optimal Target Finder were cloned into pU6-BbsI-chiRNA (a gift from Melissa Harrison, Kate O'Connor-Giles, and Jill Wildonger). A combination of two sgRNA-containing plasmids were injected into the yw; nos-Cas9(y+)/CyO strain by Genetivision Corp to delete portions of the *Csgalnact* gene and repair by nonhomologous end joining. A single sgRNA sequence was used to generate small deletions in the *C4st* protein coding sequence. Resultant deletions were screened *via* PCR, verified by Sanger sequencing, followed by backcrossing with Oregon-R strain for five generations. The sgRNA sequences used are shown in Figs. S1 and S2.

### Preparation of adult wings

The right wings from female flies were dehydrated in ethanol and subsequently with xylene (17, 44). The specimens were mounted in Canada balsam (Benz Microscope).

### Immunohistochemistry and immunoblot analysis

Immunostaining of the wing discs was performed as previously described (19, 43). Mouse anti-CS A (1:100 dilution; Tokyo Chemical Industry, LY111) was used to detect CS. Alexa488-, Alexa568-, and Alexa633-conjugated secondary antibodies (Thermo Fisher Scientific) were used at a dilution of 1:200. Images were obtained using a Zeiss 710 laser scanning confocal microscope.

For immunoblot analysis, protein samples were extracted from adult flies by SDS sample buffer. Mouse anti-CS A (1:1000 dilution; Tokyo Chemical Industry, LY111) and mouse anti-αTubulin antibody (1:2000 dilution; Sigma–Aldrich, DM1A) were used as primary antibodies. Signals were detected using horseradish peroxidase–conjugated secondary antibodies and Pierce ECL Western Blotting Substrate (Thermo Scientific).

### Disaccharide analysis

CS isolation and disaccharide composition analysis were carried out as previously described (45, 46, 47, 48, 49). Twenty adult flies or 50 brains of third instar larvae were used to isolate CS. The CS sample was digested with chondroitinase ABC (1 mU; EC4.2.2.4, Sigma–Aldrich), and the resulting disaccharide species were separated using reversed-phase ion-pair chromatography (Docosil C22 [4.6 × 150 mm; particle size, 5 μm], Senshu Scientific).

### Gel-filtration chromatography of CS

To measure the CS chain length, the purified glycosaminoglycan fraction was subjected to reductive β-elimination using NaBH_4_–NaOH and then analyzed by gel-filtration chromatography on a Superose 12 10/300 GL column (10 mm × 300 mm) eluted with 0.2 M ammonium bicarbonate at a flow rate of 0.35 ml/min. Fractions were collected at 0.5 min intervals starting at 20 min, freeze-dried, and digested with chondroitinase ABC. The digests were separated using reversed-phase ion-pair chromatography, as described previously.

### GalNAc transferase assay of human and *Drosophila* Csgalnact

A complementary DNA clone of *Drosophila Csgalnact* (NM_001299348) was obtained from GenScript. To obtain *a soluble form of* Csgalnact protein, a truncated fragment of *Csgalnact* complementary DNA, lacking the first 31 amino-terminal amino acid residues containing the putative cytoplasmic and transmembrane domains, was amplified by PCR using a 5′-primer (5′-GCGGAATTCCGGATTCGATGAACTGACGAC-3′) containing an in-frame EcoRI site and a 3′-primer (5′-AGAGGATCCTCACGATGTCATCTTTGCCGC-3′) containing a BamHI site and stop codon. The PCR fragment was subcloned into p3xFLAG-CMV8 (Sigma–Aldrich). An expression plasmid of human *CSGALNACT1*, p3xFLAG-CMV8/hCSGALNACT1, was gifted by Shuhei Yamada and Shuji Mizumoto (8, 30).

Human *CSGALNACT1* or *Drosophila Csgalnact* was transfected into 293T cells on 100-mm plates using FuGENETM 6 (Roche Applied Science). Two days after transfection, 1 ml of the culture medium was collected and incubated with anti-FLAG affinity agarose resin (Wako) for 12 h at 4 °C. The beads were recovered by centrifugation and washed with the assay buffer. The recovery of both recombinant enzymes was confirmed by immunoblot analysis using an anti-FLAG antibody (Wako).

The GalNAc-transferase assay was performed as described previously (8, 22, 30). The assay mixture contains 10 μl of enzyme-bound anti-FLAG affinity resins, 50 mM 2-(*N*-morpholino) ethanesulfonic acid–NaOH (pH 6.5), 10 mM MnCl_2_, 10 mM MgCl_2_, 0.1 mM uridine diphosphate–GalNAc as the sugar donor substrate, and 2 mM GlcA-MU (Sigma–Aldrich) as the sugar acceptor in a total volume of 50 μl. The mixtures were incubated for 12 h at 37 °C. The products of the reactions on GlcA-MU were isolated by gel filtration on an Asahipac GS-320 HQ column (7.5 mm × 300 mm) eluted with 6 mM NH_4_H_2_PO_4_ (pH 6.5)/15% CH_3_CN_3_ at a flow rate of 0.25 ml/min. The effluent was monitored fluorometrically (excitation 325 nm, emission 380 nm).

### Behavioral assays

Larval locomotion assay was performed as previously described (33). Briefly, a larva was placed at the center of 15 cm agar plate, and 45-s movies of the larva crawling were captured on an iPhone v.12.0 mini. Movies were converted into image sequences, and the locomotion of each larva was manually tracked using the Manual Tracking plugin on Fiji (National Institutes of Health). Average velocity of each animal was calculated from the movement of the larva over 44 frames. Approximately eight larvae of each genotype were tracked, and one-way ANOVA with Dunnett's test and two-sided, unpaired *t* tests were used to calculate *p* values.

Adult locomotion was analyzed by negative geotaxis assay (34, 35). Age-synchronized cohorts of flies were transferred without anesthesia to a 50 ml glass cylinder, tapped to the bottom with cotton. After a period of adaptation of 30 s, the climbing ability (climbing index) of flies was quantified as number of animals that reached the top of the cylinder (10 cm) in 15 s. Flies were assayed in batches of 20 (1:1 female/male ratio), and the test was repeated three times for each batch of animals. More than 120 flies were tested for each genotype. The number of top climbing flies was converted into percent value, and the mean percent value (±SEM) was calculated for at least six experiments.

## Data availability

All data are contained within the article.

## Supporting information

This article contains supporting information.

## Conflict of interest

The authors declare that they have no conflicts of interest with the contents of this article.
