# Supplementary material for: In vivo analysis of Drosophila chondroitin sulfate biosynthetic genes
Source: J Biol Chem. 2025 Oct 7;301(11):110783. doi: 10.1016/j.jbc.2025.110783 (PMC12630343; doi:10.1016/j.jbc.2025.110783)
Supplement: Supplemental Materials [file mmc1.docx]

**A**

5'-GCCCTCGATAAAGTCATCCAGGG-3'

5'-GATAATCAGGGCCACCGACCCGG-3'

**B**

1 MHNSLVTKLLLRLLLLFALLTGASIVVLTKCGFDELTTRETQANNPSESS

51 GFSYTLSEREAEIERLKQEVLALRTQILFLQNNRSTAKPSNGSLQLQETT

101 AGPPTAPLGHHYDCSSYIRKQVGAAEILHGLPLNNEYELIPYNHFTFTRV

151 YPIDLGLGKRVVEKPIGYRRRDLIEAVNKALESLNRNHSARIRAKGAGSA

201 AAYASDVIKYTLDDFIEGIYRNEPTTGTQYELYFQSVKHQASPVRRALVM

251 RPFAPLQTVQLSELSSSVDNSGAPPSHSPPIIHVILPLAGRLHSFRGFLQ
301 MFAKLEDRRLELIVVYFGTSGLEQARSLAGRSQRTQFLALNETFSRAKAL

351 RLGAEHIQPAEEDVLLFMCDVDIMFTTKFLERCRWNAAPGKKVYYPVVFS

401 LYNPHVVYSLQGKPLPSEEEQLVISRDTGFWRDFGYGMTCQYRSNFLKVR

451 GFDEEEIVGWGGEDVMLYRKYVRSKIKIIRATDPGIFHRWHTKICSSSLT

501 ADQYRACIRSRALNEASHAQLGFLAFRDDIAAANAAKMTS

**Figure S1. Generation of *Csgalnact^212^* allele.**

(A) The gRNAs sequences. The CRISPR/Cas9-mediated mutagenesis induced a deletion of 1018 base pairs extending from the first to the third exons and an insertion of 11 base pairs (GTACAACGATG) of unknown origin. This allele was referred to as *Csgalnact^212^*. (B) The entire amino acid sequence of wild-type Csgalnact (540 amino acids) is shown. In *Csgalnact^212^*, the 1018 bp-deletion and 11 bp-insertion caused a frame shift, resulting in a truncated protein. The truncated protein contains the first 207 amino acid residues of the wild-type protein (underlined) followed by additional 34 residues.

**A**

5'-GGATCCCTGGCCCACTCCGTGGG-3'

**B**

1 MDQLVWLSFCISIGFSLLGNFSTGLPFEDGNNNNVKITRRSLELTQTINI

51 QRQEFMQRQCELLGDHTQTLEDLSELQMDHMIVDKEHKLLYCYVPKVACT

101 NWKRVLMMLTNKWHNGTDPLQIPGSLAHSVGMFTKLYDLSEAEQQQVLSD

151 EYTRFILVRHPLERLLSAYRNKLEGDSPSARYFQSRVGRQIVKELRPGAS

201 NNSLERGDDVSFGEFIQYLVTPELSRANQSDYNEHWEVIAKLCNPCVMKY

251 NVVGKYDTLLDDSALALYLAGADNLTFPTGHKPSSTRANLRNYFDPLPIG
301 AIRKLYDIYEDDFRLFDYALDEVLGFEFG

**Figure S2. Generation of *C4st^340^* allele.**

(A) The gRNA sequence. The CRISPR/Cas9-mediated mutagenesis induced a deletion of 1 base pair (C, underlined). This allele was referred to as *C4st^340^*. (B) The entire amino acid sequence of wild-type C4ST (329 amino acids) is shown. In *C4st^340^*, "C" at nucleotide position of 387 is deleted. This 1 bp-deletion caused a frame shift, resulting in a truncated protein (amino acid number 1-129, underlined) followed by additional 62 residues.


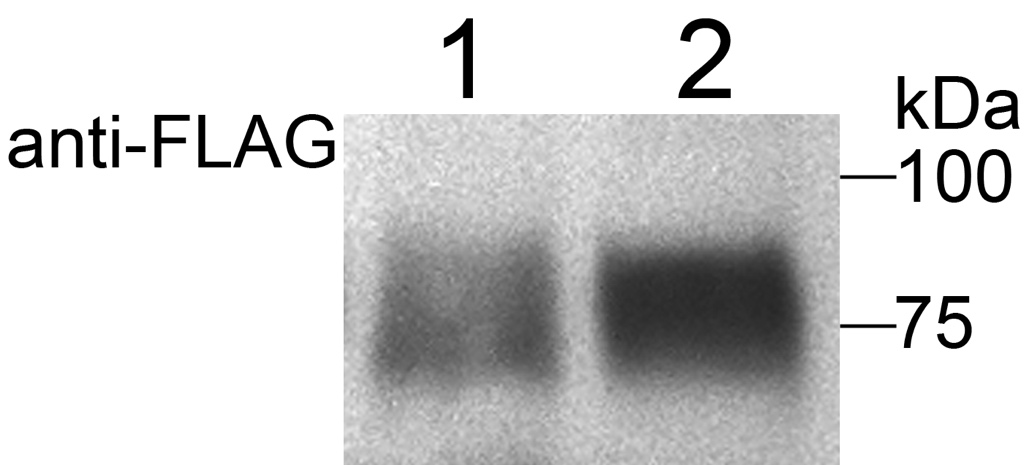


**Figure S3. Immunoblot analysis of human CSGALNACT1 and *Drosophila* Csgalnact.**

Soluble forms of human CSGALNACT1 and *Drosophila* Csgalnact with a FLAG epitope tag were expressed in 293T cells. The [recombinant proteins](https://www.sciencedirect.com/topics/biochemistry-genetics-and-molecular-biology/recombinant-protein) secreted in the medium were pulled down from the conditioned media using anti-FLAG affinity agarose. The recovered proteins were analyzed by immunoblot analysis using anti-FLAG antibody. Lane 1, human CSGALNACT1-FLAG; Lane 2, *Drosophila* Csgalnact-FLAG.
